# Base editing in the AUTS2 gene and high-throughput NGS genotyping of clones: a strategy for generating a cellular model

**DOI:** 10.18699/vjgb-26-04

**Published:** 2026-03

**Authors:** A.P. Yan, P.A. Salnikov, А.А. Buzdin, V.А. Кovalskaia, E.V. Musatova, P.S. Orlov, О.P. Ryzhkova, A.I. Subbotovskaia, М.V. Suntsova, А.U. Khristichenko, А.А. Khabarova

**Affiliations:** Institute of Cytology and Genetics of the Siberian Branch of the Russian Academy of Sciences, Novosibirsk, Russia Novosibirsk State University, Novosibirsk, Russia Sirius University of Science and Technology, Sirius Federal Territory, Krasnodar region, Russia; Institute of Cytology and Genetics of the Siberian Branch of the Russian Academy of Sciences, Novosibirsk, Russia Novosibirsk State University, Novosibirsk, Russia; I.M. Sechenov First Moscow State Medical University of the Ministry of Healthcare of the Russian Federation, Moscow, Russia National Medical Research Center for Endocrinology named after Academician I.I. Dedov, Moscow, Russia M.M. Shemyakin–Yu.A. Ovchinnikov Institute of Bioorganic Chemistry of the Russian Academy of Sciences, Moscow, Russia; Research Centre for Medical Genetics, Moscow, Russia; Center of Genetics and Reproductive Medicine “Genetico”, Moscow, Russia; Institute of Cytology and Genetics of the Siberian Branch of the Russian Academy of Sciences, Novosibirsk, Russia Federal Research Center of Fundamental and Translational Medicine, Novosibirsk, Russia; Research Centre for Medical Genetics, Moscow, RussiaA.I. Subbotovskaia; Federal Research Center of Fundamental and Translational Medicine, Novosibirsk, Russia; I.M. Sechenov First Moscow State Medical University of the Ministry of Healthcare of the Russian Federation, Moscow, Russia National Medical Research Center for Endocrinology named after Academician I.I. Dedov, Moscow, Russia; National Medical Research Center for Endocrinology named after Academician I.I. Dedov, Moscow, Russia; Institute of Cytology and Genetics of the Siberian Branch of the Russian Academy of Sciences, Novosibirsk, Russia

**Keywords:** induced pluripotent stem cells (iPSCs), CRISPR/Cas9, single-nucleotide substitutions, allele-specific expression analysis, chromosomal rearrangement, индуцированные плюрипотентные стволовые клетки (ИПСК), CRISPR/Cas9, однонуклеотидные замены, аллель-специфичная оценка экспрессии, хромосомная перестройка

## Abstract

Studying the molecular mechanisms underlying autism spectrum disorders (ASD) requires cellular models capable of capturing cis-regulatory effects and allele-specific gene expression. In this study, we present an approach for generating induced pluripotent stem cells (iPSCs) modified using an adenine base editor (ABE) to introduce synonymous single-nucleotide substitutions in the AUTS2 gene – a candidate involved in ASD pathogenesis. These substitutions serve as allele-specific markers, enabling the tracking of expression differences between normal and rearranged alleles in a cis-regulatory context. We developed a high-efficiency strategy for genotyping clones using amplicon-based next-generation sequencing (NGS). Analysis of over 100 subclones demonstrated that this approach surpasses Sanger sequencing in scalability, sensitivity, and cost-effectiveness. We selected clones with targeted heterozygous substitutions, assessed mosaicism levels, and performed phasing with germline heterozygous variants to confirm monoclonal origin and identify the allele carrying the chromosomal rearrangement. The resulting iPSC lines mark distinct AUTS2 alleles, providing a foundation for analyzing the impact of cis-regulatory elements on gene expression across different cell types. Our findings highlight the practical value of base editors and targeted NGS genotyping in creating cellular models with single-nucleotide substitutions for both basic and applied research.

## Abstract (ru)

Изучение молекулярных механизмов, лежащих в основе расстройств аутистического спектра (РАС), требует создания клеточных моделей, способных отражать цис-регуляторные эффекты и аллель-специфичную экспрессию генов. В настоящем исследовании мы представляем подход к получению индуцированных плюрипотентных стволовых клеток (ИПСК), модифицированных с использованием аденинового редактора оснований (ABE), для введения синонимичных однонуклеотидных замен в ген AUTS2 – кандидата на участие в патогенезе РАС. Эти замены позволяют маркировать аллели и отслеживать различия в экспрессии нормального и реорганизованного аллелей в цис-контексте. Мы разработали стратегию высокоэффективного генотипирования клонов с использованием секвенирования продуктов ПЦР (ампликонов) на платформе нового поколения (NGS). Анализ более 100 субклонов показал, что предложенный подход превосходит секвенирование по Сэнгеру по масштабируемости, чувствительности и экономичности. Мы отобрали клоны с целевыми гетерозиготными заменами, оценили уровень мозаицизма и провели фазирование с герминальными гетерозиготными вариантами, позволяющее убедиться в моноклональном происхождении клеточной линии или идентифицировать аллель, ассоциированный с мутацией. Полученные линии ИПСК маркируют разные аллели гена AUTS2, что открывает перспективу анализа влияния цис-регуляторных элементов на экспрессию гена в различных типах клеток. Результаты работы подчеркивают практическую значимость редакторов оснований и целевого NGS-генотипирования при создании клеточных моделей с однонуклеотидными заменами для фундаментальных и прикладных исследований.

## Introduction

According to Global Burden of Disease 2021 data, approximately 61.8 million people worldwide lived with autism spectrum disorders, with cumulative disabilityadjusted life years lost totaling 11.5 million – equivalent to 147 years per 100,000 people (Global Burden of Disease Study 2021…, 2025). Investigating the genetic causes of autism, like other congenital neurological disorders, often requires studying pathological processes occurring in brain tissue. Given the limited availability of this organ for direct cell sampling, establishing cellular models to monitor changes in target gene expression is especially important. iPSCs derived from various patient cell types serve as indispensable tools, as they can be directed to differentiate into multiple lineages, providing flexibility for modeling processes across different tissues (Rowe, Daley, 2019; De Masi et al., 2020).

CRISPR/Cas9 is widely used to recreate pathogenic mutations in cellular models. However, the system has notable limitations, including off-target activity and the imprecise repair of double-strand breaks, both of which can introduce unintended mutations (Smirnov et al., 2016; Uddin et al., 2020). To overcome these drawbacks, more precise CRISPR/Cas9-based technologies are currently being developed. One such approach is base editing, which enables specific nucleotide substitutions (A→G or C→T) without generating double-strand breaks (Gaudelli et al., 2017).

Introducing modifications with single-nucleotide precision enables not only modeling genetic diseases caused by point mutations (Lu, Huang, 2018; Geurts et al., 2023), but also allele labeling, providing a unique opportunity to trace the impact of cis-regulatory variants on gene expression and functional activity at the monoallelic level. Comparing allele expression levels allows the assessment of cis-regulatory effects while neutralizing any trans-influences (Salnikov et al., 2024). Additionally, base editors are actively used for developing gene therapy strategies for diseases associated with variants in nuclear and mitochondrial DNA (Billon et al., 2017; Liang et al., 2023).

The base editing system uses a hybrid protein consisting of nuclease-inactive Cas9 and a nucleotide deaminase enzyme. This chimeric protein enables A→G or C→T substitutions without creating double-strand DNA breaks. For the adenine base editor (ABE), deamination occurs on one DNA strand within a narrow deaminase activity window (four nucleotides) while a single-strand nick on the other strand stimulates the repair system to restore the sequence according to the modified strand (Rees, Liu, 2018). The ABE system induces A→G conversion by deaminating adenine to hypoxanthine, which DNA polymerase then recognizes as guanine (Gaudelli et al., 2017; Chen et al., 2023).

Thus, base editors catalyze direct chemical nucleotide conversion without double-strand breaks, minimizing chromosomal aberration risk and eliminating the need for exogenous DNA delivery as a homology-directed repair template. This substantially reduces off-target insertions, deletions, and chromosomal rearrangements compared to classical Cas9.

Despite the obvious advantages of this editing system, base editors have several limitations in application. A key drawback is the catalytic specificity of deaminase enzymes, enabling only transitions (C→T or A→G; purine-to-purine or pyrimidine-to-pyrimidine substitutions), but not transversions (purine-to-pyrimidine or vice versa), significantly narrowing the spectrum of correctable mutations (Komor et al., 2016; Gaudelli et al., 2017; Rees, Liu, 2018). Another serious issue is off-target activity, manifesting both as editing of nonfully complementary DNA sites within the “editing window” and nonspecific cellular RNA modification. Deaminases can randomly deaminate cytosines in RNA, potentially disrupting the transcriptome and causing toxic effects (Grünewald et al., 2019; Jin et al., 2019; Zuo et al., 2019; Yu et al., 2020).

This article focuses on using the ABE base editing system and an innovative cellular clone genotyping strategy for creating iPSC-based cellular models. Karyotype analysis of an autism spectrum disorder patient revealed a chromosomal rearrangement on one allele, with its boundary in an intergenic region near the AUTS2 gene (Gridina et al., 2025). We hypothesize that this rearrangement may disrupt AUTS2 expression regulation and potentially be linked to the patient’s phenotype. To experimentally test this hypothesis, a model is needed to assess expression differences between the normal allele and the one in cis with the chromosomal rearrangement across cell types. However, no single-nucleotide substitutions were found in this patient’s AUTS2 coding region to discriminate transcripts from different alleles. From previously obtained iPSCs of this patient, using the ABE system, we generated model lines carrying heterozygous single-nucleotide substitutions in exon 10 of the AUTS2 gene. These lines enable obtaining neurons/ neural precursors in vitro and quantitatively assessing allele-specific AUTS2 transcription from rearranged vs. intact chromosomes.

**Fig. 1.**
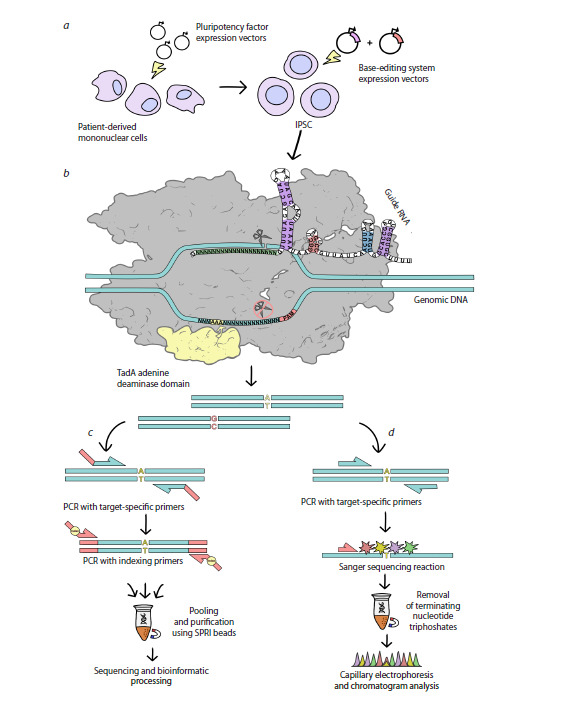
General workflow for generating single-nucleotide substitutions in human iPSCs and comparison of clone genotyping protocols using NGS and Sanger sequencing a – electroporation of patient-derived blood monocytes with reprogramming factor vectors to obtain iPSCs, followed by electroporation of iPSCs with plasmids encoding the base editing system: the target sgRNA and a modified Cas9 fused to the TadA adenine deaminase; b – schematic representation of the complex of modified Cas9 (gray), fused to TadA (light yellow), with an sgRNA and protospacer sequence on genomic DNA. Adenines within the editing window are highlighted in yellow; c – NGS library preparation scheme using addition of adapter sequences and indexing via two rounds of PCR; d – standard Sanger sequencing workflow.

Clone genotyping and editing efficiency assessment are routinely performed by Sanger sequencing of target locus amplicons. The most convenient and accurate approach is sequencing the amplicon containing the target substitution. For amplicon sequencing, two methods can be applied: Sanger and NGS (Fig. 1c, d). The choice between Sanger and NGS depends on specific research tasks. Sanger remains optimal for analyzing individual PCR products with few samples. Its limited throughput makes it economically unfeasible for large sample sets. In contrast, NGS technologies enable simultaneous sequencing of numerous samples, significantly reducing per-sample analysis cost and making the method indispensable for high-throughput analysis. However, NGS requires bioinformatics. Thus, for routine single amplicon analysis Sanger retains advantages, while for scaling analysis to large sample numbers NGS demonstrates clear superiority.

In this study, NGS analysis of 117 subclones identified cell lines suitable for differentiation and AUTS2 expression analysis. We provide a detailed protocol for NGS genotyping sample preparation and bioinformatics analysis of obtained data, adaptable for other geno- typing tasks.

## Materials and methods

Creation of the MLM3636-BstV2I vector. The base vector was plasmid MLM3636 (Addgene #43860), containing a guide RNA (gRNA) sequence with a spacer cloning site designed for BsmBI restriction enzyme (New England Biolabs, USA). The following protospacer sequences were used: 5′-CAAAAGTTGACCCATTC TAC-3′ for N044 and 5′-TCACACTGTGCCGGTA GAAT-3′ for N068. Since this enzyme is not produced in Russia, to simplify subsequent manipulations, we replaced the original cloning site with an analogous site from plasmid pSpCas9(BB)-2A-GFP (PX458) (Addgene #48138), recognized by BstV2I enzyme produced by the Russian manufacturer SibEnzyme LLC.

For this, we amplified the fragment containing the pSpCas9(BB)-2A-GFP (PX458) cloning site using primers pSpCas9_clonF and pSpCas9_clonR, and nearly the entire MLM3636 vector sequence using primers MLM3636_clonF and MLM3636_clonR. The pro- duct was treated with 1 U DpnI enzyme (New England Biolabs, USA) and purified using SPRI bead suspension VAHTS DNA Clean Beads (Vazyme, China). Assembly was performed using NEBuilder HiFi (New England Biolabs, USA) according to the manufacturer’s protocol.

To obtain vectors expressing gRNAs, 1 μg of MLM3636-BstV2I plasmid was linearized with 25 U BstV2I enzyme in a 50 μl reaction mixture at 55 °C overnight. After restriction, DNA was purified using 0.8× volume of SPRI bead suspension VAHTS DNA Clean Beads (Vazyme, China). Single-stranded oligonucleotides N044_F and N044_R (2 μM each) were phosphorylated in 1× T4 DNA ligase buffer with 100 nM ATP and 10 U T4 polynucleotide kinase (SibEnzyme, Russia). The same procedure was applied for the N068 pair. Oligonucleotides were then heated to 95 °C and hybridized by slow cooling at 0.1 °C/s to 4 °C.

The resulting double-stranded oligonucleotide N044 (N068) F+R solution was ligated into the linearized MLM3636-BstV2I vector backbone. Components mixed on ice were: 100 nM ds-oligo solution, up to 20 ng previously linearized plasmid, 100 nM ATP, 1× T4 DNA ligase SE buffer, 20 U T4 DNA ligase. The reaction mixture was incubated overnight at 4 °C. The product was purified using 0.8× volume of SPRI bead suspension VAHTS DNA Clean Beads (Vazyme, China) and eluted in 5 μl H2O. Half the product volume was used for subsequent bacterial cell transformation.

Escherichia coli transformation was performed by electroporation, plasmid DNA was isolated using Midi-prep kit (Evrogen, Russia), and additionally purified using 0.8× volume of VAHTS DNA Clean Beads (Vazyme, China). Elution was performed in a minimal water volume to achieve DNA concentration ≥1 μg/μl. For puromycin selection, plasmid pURC_puro (provided by A. Nurislamov) was used. This construct expresses a puromycin resistance gene under a CMV promoter integrated into the plasmid, enabling selection and enrichment of the cell population for editing events.

iPSC culture and neon electroporation. iPSCs were cultured on plastic plates pre-coated with Matrigel cultural matrix solution (Corning, USA) using mTeSR1 medium (StemCell, Canada) supplemented with penicillin/ streptomycin antibiotic mixture (PanEco, Russia). Cells were dissociated using TripLE reagent (Thermo- Fisher, USA) and passaged at a 1:3–1:5 ratio with the addition of the Y-27632 inhibitor (10 μM, Rho-kinase inhibitor) (StemCell, Canada). The day before electroporation, iPSC lines were passaged to achieve ≤70 % confluency. Before transferring transfected cells, culture plate bottoms were coated with Matrigel matrix (1 ml per 10 cm2) and incubated at 37 °C for 20–30 minutes. 1 hour before transformation, 10 μM Y-27632 was added to cells.

Cell culture was dissociated using 700 μl TripLE (ThermoFisher, USA). 1 ml wash medium was added per well, cells were gently resuspended, and the suspension was collected into a centrifuge tube. Cell counting was performed using a hemocytometer. Suspension was centrifuged at 300 g for 5 min. After counting, the required cell suspension volume (150–200 thousand cells per 1 electroporation reaction) was washed once in phosphate buffer. In a 1,500 μl tube, three plasmids were mixed (0.5 μg each): gRNA expression plasmid (MLM3636-BstV2I), base editor (Addgene #108382), and pURC_puro (puromycin resistance). 150–250 thousand cells, resuspended after washing in phosphate buffer, were added into Neon electroporation buffer R. Total cell suspension and plasmid mixture volume was not supposed to exceed 10 μl.

Electroporation was performed using Neon system at 1,100 V – 30 ms – 1 pulse, and cells were transferred to a 6-well culture plate well in 3 ml antibiotic-free mTeSR1 medium. After 24 hours, antibiotics (1× penicillin/streptomycin mixture) and selective antibiotic puromycin (1 μg/ml for 48 hours) (Gibco, USA) were added to the medium. The medium was fully changed after 1 day. At 7–10 days, individual cell colonies were manually transferred to separate wells of a 24-well plate (~30 colonies picked). When colonies reached 1/4 well area, they were dissociated using TrypLE, and after centrifugation, part of the cell suspension was used for DNA extraction and subsequent genotyping, while the remainder was frozen in KSR (Gibco, USA) with 10 % DMSO.

Clone genotyping using amplicon NGS sequencing. PCR setup used reagents from MBS-Technology (Russia). Total 50 μl volume contained: 50 ng DNA, 1× MBUsion buffer, 0.2 mM of each dNTP, 0.2 μM AUTS_gen_F primer, 0.2 μM AUTS_gen_R primer (Table 1), 0.4 μl MBUsion polymerase (MBS, Russia), H2O to 50 μl. PCR conditions were as follows: 95 °C for 3 min, 20 cycles: 95 °C for 15 s, 60 °C for 30 s, 72 °C for 30 s, then 72 °C for 1 min, 4 °C. PCR products were diluted to 1:100, and 2 μl was used as a template for indexed PCR including full sequencing-required sequences P5_IP{index_ID} and P7_IP{index_ID} (Table 1).

**Table 1.**
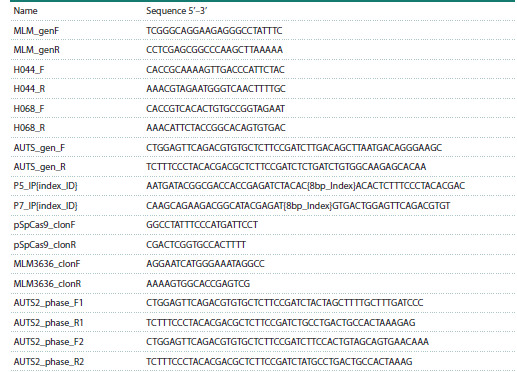
Primers and oligonucleotides

Amplification conditions were: 95 °C for 3 min, 8 cycles: 95 °C for 15 s, 60 °C for 30 s, 72 °C for 30 s, then 72 °C for 1 min, 4 °C. PCR product concentrations were approximately assessed by agarose gel electrophoresis visualization, samples were pooled in equal amounts, purified using 1 volume VAHTS DNA Clean Beads (Vazyme, China), and eluted in 20 μl H2O.

Sequencing was performed in paired-end 150 bp mode (~10,000 reads per sample). Adapter sequences were removed (cutadapt) and aligned to indexed target region reference sequence using bowtie2 (default parameters). BAM files were visualized in Integrative Genomics- Viewer (IGV, USA).

Variant identification used bcftools mpileup with allele depth recording (AD flag). A custom Python script calculated mutant allele frequency per position, aggregated results into matrix, and generated line graphs for sample groups to assess allele frequency distribution across all possible substitution positions. All scripts with examples are available on GitHub: https://github.com/Somatich/ NGS-genotyping-Yan-et-al.-2025.

Phasing introduced substitutions with a germline variant. Phasing of single-nucleotide substitutions determines whether two variants are in cis or trans configuration. We designed primer pairs flanking both induced mutagenesis site and germline variant (chr7:70768282 hg38, rs3829006 G/A), yielding 363 bp of PCR product; 5′-ends included NGS technical sequences. This germline variant is 238 bp from the mutagenesis site, selected from patient exome sequencing data (Gridina et al., 2025). Distance ≤200–300 nucleotides yields optimal PCR product length for Illumina NGS sequencing (DNA fragments >500 bp significantly reduce efficiency). Two primer pairs were used (AUTS2_phase_F1/R1 and AUTS2_phase_F2/R2) (Table 1).

PCR setup used MBS-Technology reagents (Russia) as described above. Indexed PCR and cleanup were performed identically. Libraries were sequenced paired-end (150 bp), yielding ~5,000 read pairs per sample. Reads were mapped using Bowtie2 (default settings). SNP phasing was performed manually by analyzing alignments in the IGV genome browser.

## Results

**Design of sgRNAs and evaluation of editing efficiency in exon 10 of the AUTS2 gene**

The aim of this study was to generate an iPSC-based cellular model carrying a heterozygous synonymous SNV in exon 10 of the AUTS2 gene using adenine base editing (ABE) (Fig. 1a). Exon 10 was selected because it is present in most of the described and predicted transcripts; moreover, a germline single-nucleotide variant located near exon 10 enables phasing of the introduced substitution with respect to alleles.

We identified adenine positions located at the third codon positions and falling within the active ABE editing window (nucleotides 4–7 of the protospacer). Thus, A→G substitutions at these positions are synonymous. Two such adenines were identified: chr7:70768044 A→G and chr7:70768068 T→C (hg38). The chr7:70768044 A→G substitution yields a synonymous AAA→AAG change (p.Lys570Lys). This variant occurs in the population with a frequency of 0.000005596 and is not predicted to affect splicing or create a novel splice site (SpliceAI, MobiDetails). The chr7:70768068 T→C substitution is likewise synonymous (AGT→AGC), is absent from gnomAD, and is also not predicted to influence splicing (SpliceAI, MobiDetails) (Reese et al., 1997; Jaganathan et al., 2019; Baux et al., 2021).

For both candidate sites, two guide RNAs – H044 and H068 – were designed (Table 1). Their in vivo activity was tested in iPSC culture using a plasmid expressing Cas9 (Addgene #62988). Both sgRNAs demonstrated comparable editing efficiencies: 9.2 % for H044 at chr7:70768044 and 7.6 % for H068 at chr7:70768068.

Editing outcomes were analyzed using Cas-Analyser (Crisper Rgen) (Hwang et al., 2018). It is important to emphasize that genome editing efficiency is determined by multiple poorly controllable factors, such as the local chromatin environment in a given cell line. Therefore, computational predictions of sgRNA activity and specificity cannot be fully relied upon. To increase the likelihood of obtaining the desired mutation, it is reasonable to use multiple sgRNAs directed at the same region. Based on this rationale, both sgRNAs were carried forward into subsequent experiments.

As a pilot experiment, we assessed the efficiency of each guide in combination with the adenine base editor Cas9(ABE7.10) in a bulk cell population (i. e., without selecting for transfected cells). Three days after electroporation with plasmids encoding Cas9 and the sgRNA, genomic DNA was extracted and libraries were prepared for NGS. BE-Analyser (Crisper Rgen, South Korea) (Hwang et al., 2018) was used for analysis. The ABEmediated editing efficiency for H044 at chr7:70768044 was 5.1 %.

Four adenines lie adjacent to the target nucleotide (chr7:70768041–70768043). Their editing frequencies were 3 % at chr7:70768043; 0.4 and 0.7 % at chr7:70768041 and chr7:70768042, respectively. For H068, the efficiency at chr7:70768068 was 3 %. These results indicate that, despite the presence of a cluster of four adenines near the target site, the H044 protospacer yields higher editing efficiency at the intended position compared to H068. Although the relatively high off-target editing frequency at chr7:70768043 was not critical for further use of H044, it necessitated screening a larger number of clones to identify lines carrying only the desired substitution.

To increase the proportion of successfully edited cells in the population, we additionally co-transfected a plasmid containing a selectable marker (puromycin resistance), enabling enrichment of transfected cells and substantially improving the yield of edited clones. Taken together, the pilot data confirm that ABE editing is functional at the AUTS2 locus. The H044 sgRNA demonstrates superior on-target efficiency relative to H068, despite potential issues associated with the adenine cluster.

**Generation and selection of iPSC clones carrying the introduced nucleotide substitution**

**Fig. 2.**
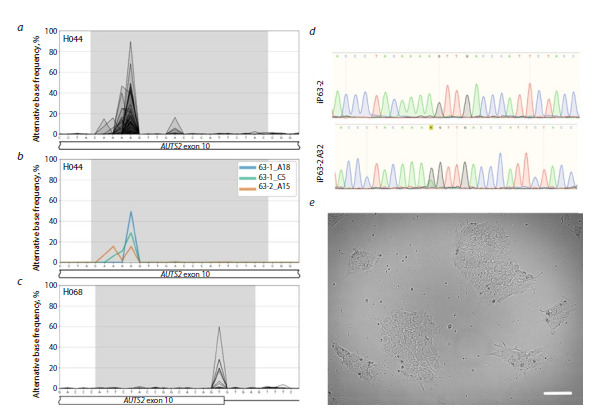
Examples of the results obtained using the presented protocol. a – visualization of the aggregated genotyping results for 69 cell clones modified using guide RNA H044; b – example of the visualization of genotyping results for three selected samples. The X axis shows the genomic sequence chr7:70768037–70768062, hg38 (in nucleotides), and the Y axis shows the percentage of sequencing reads containing a substitution; c – visualization of the aggregated genotyping results for 48 cell clones modified using guide RNA H068. The X axis shows the genomic sequence chr7:70768048–70768077, hg38. The grey area corresponds to the guide RNA sequence used in the experiment; d – sequencing chromatograms of samples iP-63-2 (the original iPSC line) and iP-63-2A32 (the iPSC line with a synonymous AAA/AAG triplet substitution) at the mutagenesis site; e – morphology of the iPSC culture in transmitted light; the white bar represents 100 μm.

Electroporation using the Neon™ system was performed to introduce the target SNV into exon 10 of AUTS2. Two patient-derived iPSC lines were electroporated with a mixture of three plasmids encoding the sgRNA, the adenine base editor and a puromycin resistance marker. After electroporation, cells were plated at low density onto matrix-coated plates and cultured in mTesR1 medium supplemented with puromycin for three days. On days 7–10, individual colonies exhibiting characteristic iPSC morphology were visually identified and manually picked (Fig. 2e). A total of 117 clones displaying stable growth and typical pluripotent morphology were retained for further analysis

**Fig. 3.**
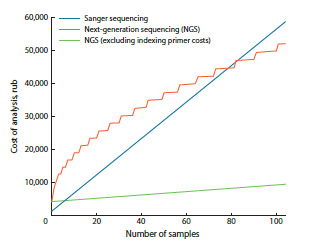
Dependence of genotyping cost by Sanger sequencing (blue) and by NGS, with (orange) and without taking into account (green) the cost of synthesizing indexing primers.

A key challenge at this stage was genotyping over 100 samples. Conventional assessment of genome editing outcomes relies on Sanger sequencing of PCR amplicons spanning the edited region (Fig. 1c, d). However, this approach becomes labor-intensive and economically inefficient when analyzing >80 samples (Fig. 3). We therefore implemented high-throughput genotyping via next-generation sequencing of amplicons. This method provides excellent scalability and dramatically accelerates analysis, allowing simultaneous determination of both allele composition and allele frequencies within each clone.

For NGS analysis, the target region harboring the substitution was amplified with site-specific primers flanking the editing site. For paired-end 150 bp reads, optimal primer placement is 50–130 bp from the mutation site (Fig. 1c), ensuring that both reads cover the edited base. Technical sequences corresponding to Illumina 3′ adapter regions were added to the 5′ ends of site-specific primers. A second PCR was performed using universal primers containing index sequences for sample pooling and demultiplexing.

**Assessment of editing efficiency and mosaicism in clones**

In total, 117 clones were analyzed: 69 edited with H044 (Fig. 2a), and 48, with H068 (Fig. 2c). Overall editing efficiency for H044 – defined as the proportion of clones with substitutions occurring at ≥2 % allele frequency – was 54 % (37/69). For downstream applications, we specifically required heterozygous substitutions; so, we filtered clones with edited allele frequencies ≥30 %. At this threshold, H044 editing efficiency was 13 % (9/69). Two clones exhibited substitution frequencies of 68 and 89 %, suggesting potential homozygosity. For H068, the overall efficiency was 31 % (15/48) at the 2 % threshold, and 4% (2/48) at the 30 % threshold.

Observed allele frequencies often deviated from the theoretically expected values (0, 50, 100 %), even in ostensibly clonal lines. Some variability likely arises from library preparation-related biases (multiple PCR cycles, cross-contamination risk). However, a major contribution likely stems from cellular cross-contamination during subcloning, as imperfect dissociation, reaggregation, and migration of cells during early culture can occur. Additional variability is introduced by asynchronous editing events: between transfection and onset of editor expression, some cells may divide, giving rise to subpopulations with different mutations or no mutations, producing mosaic colonies. Therefore, regardless of initial editing efficiency, screening a large number of clones is essential to exclude mosaic lines

Within the editing window (protospacer positions 4–6), multiple adenines were present. Substitutions occurred at all “editable” positions but with varying efficiency: the highest frequency was observed at position 5 (chr7:70768044; ~45 % positive clones at ≥2 % threshold), approximately twice the frequency of position 4 (chr7:70768043; ~17 %) (Fig. 2a). Position 6 harbors a guanine and therefore cannot be assessed, although it falls within the theoretical window. Editing frequencies at other adenines in the region did not exceed 5 %. Notably, no cases of simultaneous double substitutions within the same allele were detected, nor were T→C conversions observed on the non-target strand.

Figure 2 illustrates the visualization strategy used for presenting these results (Fig. 2a–c) – a convenient method for rapid comparison of samples, noise assessment, and identification of candidate clones for further work.

We selected five colonies carrying appropriate substitutions and displaying minimal mosaicism (<5 % mosaic alleles). To determine which allele carried the introduced substitution, as well as to assess clone monoclonality, phasing was performed with a germline heterozygous variant in the surrounding region (SNV chr7:70768282 hg38, rs3829006 G/A), previously identified by whole-genome sequencing and marking the allele in cis with the structural variant. Three non-mosaic clones were selected; two of them carried a synonymous SNV in a heterozygous state, each on a different allele (Table 2).

**Table 2.**
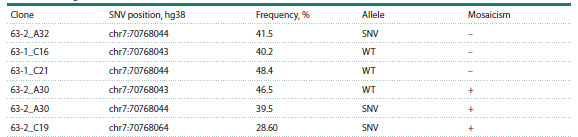
Phasing and assessment of mosaicism in AUTS2-edited colonies Note. WT – wild type, SNV – single nucleotide variant.

After confirming the absence of mosaicism, these iPSC lines were expanded for subsequent analysis of AUTS2 allele-specific expression. To prevent errors during large-scale culturing, heterozygous substitutions were re-validated by Sanger sequencing (Fig. 2d). Thus, we successfully generated the required iPSC lines and obtained a tool suitable for analyzing the impact of the patient’s chromosomal rearrangement on AUTS2 expression

## Discussion

In this study, we generated three genetically modified patient-derived iPSC lines carrying single-nucleotide substitutions in AUTS2 exon 10 and demonstrated the effectiveness of an NGS-based strategy for highthroughput genotyping of edited clones. The resulting lines are genetically homogeneous, derived from two independent iPSC lines from the same individual; two of them carry synonymous SNV marking different alleles. These lines are fully suitable for subsequent analysis of how the patient’s chromosomal rearrangement influences AUTS2 expression and its functional consequences

Allelic marking enables discrimination of cis-regulatory differences within the same cellular environment. This experimental design minimizes the influence of poorly controlled external factors: because both alleles are exposed to identical trans-acting regulators, any detected expression differences reflect only cis-regulatory variation. For this reason, even non-synonymous substitutions can be used, since any feedback-mediated effect on transcription would affect both alleles equally and thus would not distort cis-regulation assessment.

The application of NGS for genotyping significantly reduces laboratory handling time and simplifies the analysis process compared to first-generation sequencing. In contrast to Sanger sequencing of individual samples, NGS enables process scaling and allows the analysis of more than 100 samples in a single run (Table 3). Preparation of an NGS library requires routine DNA extraction and two rounds of PCR using primers specific to the target genomic region of interest, which also introduce the necessary technical sequences required for sequencing.

**Table 3.**
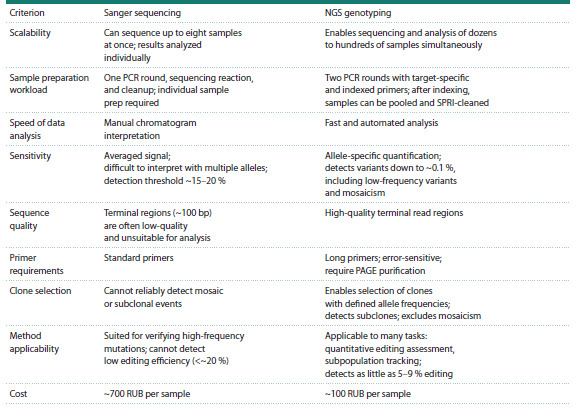
Comparison of genotyping by Sanger sequencing and by NGS

The integration of NGS with bioinformatics tools enables automated identification of nucleotide variants and eliminates errors associated with chromatogram quality in the Sanger method. NGS-based genoty- ping significantly reduces labor and simplifies analysis compared to first-generation sequencing. Unlike Sanger sequencing, which measures an averaged signal and can detect variants only at ≥20 % abundance (based on chromatogram inspection) (Davidson et al., 2012; Bennett et al., 2020), NGS provides allele-level information with detection sensitivity down to ~2 %, eliminating interpretative ambiguity.

Using this approach, we assessed sgRNA performance in bulk cell populations and identified a low modification rate (~5 %), prompting a shift to constructs with selectable markers to enrich for successfully transfected cells.Subsequent NGS analysis enabled identification of non-mosaic clones with the desired edits – critical for establishing stable genetic lines. High sequencing depth (typically ~10,000 reads per 300-bp amplicon) allows reliable detection of rare variants that are missed by less sensitive methods.

Although NGS is often perceived as prohibitively expensive, targeted amplicon sequencing reduces persample sequencing costs to <10 RUB. The main expenses are index primers, with total cost scaling as √N (where N is the number of samples). These primers are universal and compatible with both the library preparation scheme described here and standard protocols using ligated adapters. Commercial index kits are available but generally more expensive. Accounting for primer costs, NGS becomes more cost-effective than Sanger for >80 samples; without primer synthesis costs (i. e., using existing primer sets), the cost advantage appears already at >7 samples (Fig. 3; Table 4). Thus, the proposed method is highly suitable for widespread use.

**Table 4.**
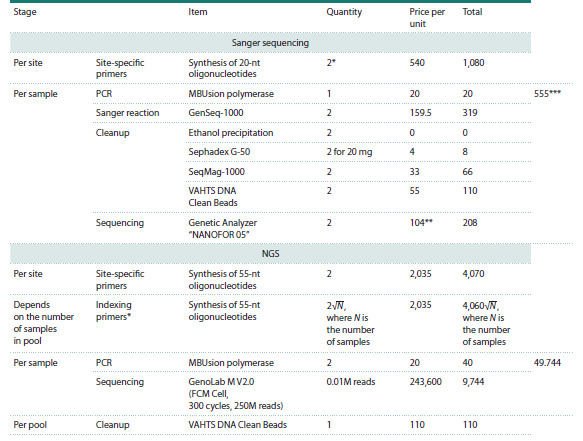
Cost estimation of sample genotyping using Sanger sequencing and NGS * Ideally, a system of four primers should be used: one pair for product generation via PCR, and two more for sequencing in both directions. ** The cost of analyzing one sample recalculated per polymer consumption in a 35-cm capillary. *** Including Sephadex G-50 cleanup.

Amplicon NGS can also be applied to assess outcomes of other CRISPR/Cas9-based editing systems. Even when induced mutations (e. g., large deletions or inversions) exceed the read length limit (~300 bp paired-end), sequencing only the mutation boundaries is sufficient to verify adjacent sequence integrity and identify undesired events. The method remains applicable as long as primer sites are intact – a limitation shared with all PCR-based genotyping.

Nonetheless, several limitations must be considered. Multiple PCR cycles can introduce amplification artifacts. Because amplicons undergo many manipulations prior to indexing and the method is highly sensitive, the risk of contamination is significant; strict post-PCR handling practices must be followed, and contact with pre-PCR areas must be avoided. Additionally, primers containing long non-genomic 5′ extensions often reduce amplification efficiency and require optimization of PCR conditions for each primer pair

## Conclusion

This article presents a method for obtaining model lines based on iPSCs with a synonymously mutated allele of the AUTS2 gene marked using base editor technology. The obtained iPSC lines will be used to study the influence of chromosomal aberrations on AUTS2 expression and to investigate the functional consequences of this impairment

The proposed approach to genotyping a large number of clones using NGS amplicon sequencing demonstrates high efficiency and scalability while reducing the cost of analysis compared to traditional methods. The high throughput of the method allowed the analysis of a large number of samples, as well as the assessment of mosaicism, composition, and allele frequencies in the cell population

Thus, the presented strategy allows highly efficient generation of cellular models for studying the influence of cis-regulatory variants on gene transcriptional activity, while controlling the genetic homogeneity of the modified cellular models, which can be in demand both in basic research and in solving applied problems.

## Conflict of interest

The authors declare no conflict of interest.
